# Detection and Characterization of Multiple Discontinuities in Cables with Time-Domain Reflectometry and Convolutional Neural Networks

**DOI:** 10.3390/s21238032

**Published:** 2021-12-01

**Authors:** Marco Scarpetta, Maurizio Spadavecchia, Francesco Adamo, Mattia Alessandro Ragolia, Nicola Giaquinto

**Affiliations:** Department of Electrical and Information Engineering, Politecnico di Bari, Via E. Orabona 4, 70125 Bari, Italy; francesco.adamo@poliba.it (F.A.); mattiaalessandro.ragolia@poliba.it (M.A.R.)

**Keywords:** time-domain reflectometry, distributed sensing, fault detection, convolutional neural network, time-domain analysis

## Abstract

In this paper, a convolutional neural network for the detection and characterization of impedance discontinuity points in cables is presented. The neural network analyzes time-domain reflectometry signals and produces a set of estimated discontinuity points, each of them characterized by a class describing the type of discontinuity, a position, and a value quantifying the entity of the impedance discontinuity. The neural network was trained using a great number of simulated signals, obtained with a transmission line simulator. The transmission line model used in simulations was calibrated using data obtained from stepped-frequency waveform reflectometry measurements, following a novel procedure presented in the paper. After the training process, the neural network model was tested on both simulated signals and measured signals, and its detection and accuracy performances were assessed. In experimental tests, where the discontinuity points were capacitive faults, the proposed method was able to correctly identify 100% of the discontinuity points, and to estimate their position and entity with a root-mean-squared error of 13 cm and 14 pF, respectively.

## 1. Introduction

Reflectometry is a technique for the detection and localization of impedance discontinuity points in electrical cables. Its principle of operation consists of transmitting a reference signal into the cable and observing the reflected signals at the same point of injection. The analysis of the reflected signals, together with additional information about the cable, yields the estimation of the position and characteristics of the discontinuity points in the cable. The first and more natural application of reflectometry is cable fault detection and localization [1], but this technique has also been used for many other measurement problems over the years. Some examples are liquid levels and properties monitoring [2], measurement of salinity and humidity in materials [3,4], measurement of complex soil dielectric permittivity [5], and skin hydration monitoring [6]. Reflectometry is also widely used in distributed monitoring applications. In this case, the cable is used as a sensing element in continuous measurements of large regions or structures. An example is the monitoring of structural elements, such as bridge steel strands [7] or concrete beams [8,9] and bored piles [10] in buildings.

In recent years, artificial neural networks have been used in many data analysis problems, among which is also the processing of reflectometry signals. The increasing adoption of these models is due to their impressive performances in performing operations that are not easily feasible using conventional signal processing techniques. In [11], an approach for fault detection and assessment in instrumentation and control cables has been proposed that makes use of time–frequency domain reflectometry (TFDR) and region-based convolutional neural networks (R-CNNs). A Gaussian envelope with a linear chirp is used as a stimulus signal in TFDR. Reflected signals measured at the cable beginning are represented in the time–frequency domain first, using the Wigner–Ville distribution. The obtained RGB images are then analyzed through the R-CNN to find the location of the reflected signals in the time–frequency domain. Additionally, a model of the reflected signals is derived from a preliminary measurement on a cable with a known length. This model is used for generating the training dataset for the neural network and for filtering out the reflected signals due to multiple reflections. The proposed technique was tested in the case of a single resistive fault in a branched network cable or in a cable without branches, while the case of multiple faults (two faults were considered) was only qualitatively analyzed. An enhanced version of TFDR (without using neural networks) is also used in [12], where both the case of localized resistive faults and of localized ohmic-capacitive faults are analyzed.

In [13], a multi-layer perceptron neural network (MLP-NN) is used for the detection of soft faults in wire networks. Since branched networks are considered, the reference signal is injected in multiple points to resolve ambiguities in the identification of branches containing faults. Before sending reflectometry data to the MLP-NN, a pre-processing step is performed. The difference between the measured reflectometry signals and reflectometry signals obtained on a healthy network is computed, and after a thresholding process, the positions and amplitudes of the peaks in the difference signals are found. These data are the input of the MLP-NN, while its outputs are the estimated positions and impedances of the detected soft faults. The usage of genetic algorithms is also explored for the same purpose, leading to accurate estimation results. An MLP-NN with TFDR is also used in [14], for fault detection in multi-core C&I cables. The aim of the method proposed in [14] is to detect the presence and position of a fault and also to differentiate the faulty line within the multi-core cable. In fact, when a line is faulty, the other lines are also affected due to crosstalk.

A generalized regression neural network (GRNN) is used in [15] for the localization of faults in rail tracks. In this case, frequency-domain reflectometry (FDR) is used for diagnosis, considering the rails as the wires of the transmission line under test. The amplitude versus frequency waveform obtained with the FDR is exploited to localize the fault, using the GRNN for non-linear regression. The type of fault (short or open) is deducted by looking at the initial inclination of the amplitude versus frequency waveform. A GRNN is also used in [16] for the analysis of features extracted from TFDR signals for shielded cables diagnosis. In particular, the purpose is to detect faults in the cable. Three features are extracted from the TFDR signals: the time delay of the reflected signal, its amplitude, and the time–frequency phase difference between the reference and the reflected signal. The GRNN uses these features as input and produces an estimate of the position and reflection coefficient of the discontinuity point. Experimental results are provided considering a single localized fault, of various entities, in the cable or at its end.

This paper develops the idea proposed in [17] by the authors, presenting a method based on time-domain reflectometry (TDR) with convolutional neural networks (CNNs) for the localization and characterization of multiple impedance discontinuity points in cables. TDR is probably the most common reflectometric method, owing to the simplicity of the stimulus signal (a narrow pulse or a short rise time step) and of the measurement process. This implies that devices for TDR are also available at a lower cost.

According to the proposed method, TDR signals are analyzed by means of a 1D-CNN that estimates the position and impedance of the discontinuity points in the cable under test. The neural network identifies all the discontinuity points, including the line termination, and hence, classification is also performed: each detected fault is associated with a class (capacitive fault or line termination).

The main advantages of our method over the others found in the literature are the following:

- No pre-processing and extraction of features from the measured signals is required. This implies less computational burden and better exploitation of the deep learning paradigm. When working directly with raw data, deep neural networks can indeed select the optimal features to extract for the given task. Additionally, a more simple and general estimation procedure is obtained;
- Accurate localization and characterization of multiple discontinuity points in the cable;
- The neural network is trained using TDR signals generated with a transmission line simulator. Even though an accurate model of the cable is required to obtain good results, once it has been created, it can be used to generate training datasets to make the neural network work in different conditions;
- The neural network can work with any cable of the same type as those used in the training set, with a variable length.

## 2. Materials and Methods

### 2.1. Measurement Setup

In this study, RG58-CU coaxial cables containing capacitive faults were studied. This setup was used as an example for developing and testing the proposed method since it was strictly controllable. However, the method can also be used for setup containing other types of cables and discontinuities, given that all the operations described in the following are not specific to the considered setup.

**Figure 1:**
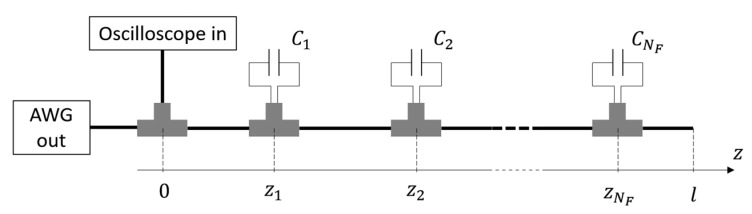
Representation of the measurement setup. Time-domain reflectometry (TDR) was applied to a cable containing $N_{F}$ parallel faults to estimate the $N_{F}$ pairs of values $z_{i}$, $C_{i}$.

The considered situation is depicted in Figure 1. The cable had total length l and contained $N_{F}$ localized faults, simulated by means of capacitors connected in parallel to the cable’s conductors through T junctions. The capacitors were placed at a distance $z_{i}$ from the beginning of the cable and had the capacity $C_{i}$. The stimulus signal was generated by an Agilent 33250A Arbitrary Waveform Generator (AWG), while the TDR signals were acquired using a LeCroy Waverunner-2 LT262 oscilloscope.

A Gaussian pulse was used as a stimulus signal since it is concentrated in both time- and frequency domains. This was useful to have cleaner reflectometry signals while having the ability to perform frequency-domain simulations such as those described in Section 2.3. The stimulus signal was therefore defined as

(1) $$v_{G}\left(t\right)=e^{-\frac{{\left(t-t_{0}\right)}^{2}}{2\sigma^{2}}}$$

where σ=8.1 ns (the lowest achievable with the hardware used for experiments) and $t_{0}=100\;\mathrm{ns}$. In experiments, a sampling frequency of 250 MHz was used for the acquisition of reflectometry signals. The number of samples acquired for each TDR signal was N=1024, corresponding to a time duration of ~4 μs. This duration was sufficient to see all the reflected signals, considering that the velocity of EM waves in RG-58CU cables is $\sim 2\times 10^{8}\;m/s$, and cables no longer than 200 m were considered.

### 2.2. Neural Network

The neural network for faults localization and characterization proposed in this article was inspired by single-shot CNNs for object detection in images [18,19]. The main component of these neural networks is a sequence of convolutional and pooling layers that have the purpose of extracting features from the images being analyzed. The level of abstraction of the extracted features increases moving toward the final layers. The result produced by this stack of convolutional layers is a set of 2D feature maps that are then further processed by densely connected layers or additional convolutional layers to finally estimate the bounding boxes containing the objects in the image and associate a class to each of them.

**Figure 2:**
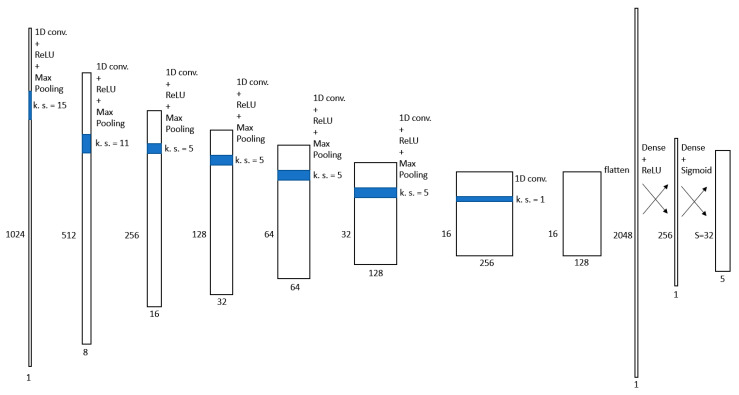
Neural network proposed for the localization and characterization of the faults.

Since time-domain signals were the object of our study, we used 1D convolutional layers instead of 2D ones as the base component of the model. The neural network for faults localization and characterization proposed in this paper is depicted in Figure 2. The input of the first layer of the neural network was the measured TDR signal that had a size of 1024×1. The first layer contained eight convolution kernels of length 15, corresponding to a time duration of 60 ns. This value was chosen to match the time duration of the stimulus signal and the reflected signals. The rectified linear unit (ReLU) was used as an activation function. The 1D max-pooling operation was then applied to the output of the convolutional layer, using a pool size of 2. The output of the neural network was therefore downscaled from 1024×8 to 512×8. The same operations (convolution, activation, pooling) were performed in five layers of the neural network, with the only difference in the number and size of convolution kernels. The five layers contain, respectively, the following:

- A total of 16 kernels of size 11×8;
- A total of 32 kernels of size 5×16;
- A total of 64 kernels of size 5×32;
- A total of 128 kernels of size 5×64;
- A total of 256 kernels of size 5×128.

The last convolutional layer had the only purpose of dimensionality reduction. Therefore, it contained 128 kernels of size 1×256, and no activation and pooling operations were performed.

This sequence of convolutional layers served to create an abstract representation of the problem. In this way, the neural network could consider accurately all the information contained in the reflectometry signal, e.g., ignoring reflected signals due to multiple reflections.

The feature maps produced by the last convolutional layer were then flattened to a 2048×1 array that was sent to the last two densely connected layers of the network. The first of them contained 256 artificial neurons and used the ReLU activation function, while the second contained 5⋅S artificial neurons and used the sigmoid activation in order to have outputs in the range (0–1). The output of the last layer was finally reshaped to build an S×5 matrix, whose rows were the predictions produced by the neural network. The range of length that could contain a discontinuity point was divided into S cells. The i-th prediction represents the estimated discontinuity point in cell i. Each prediction was an array of the following five elements:

- The first element indicated the normalized position of the discontinuity points relative to the range of lengths in the cell;
- The second and third elements indicated the class of the predicted discontinuity point. If the first of these two elements was greater than the second, a capacitive fault was predicted; an open termination of the line was predicted otherwise;
- The fourth element quantified the capacity value. This was also a normalized value relative to the range of possible capacity values. If a line termination was predicted, this value was neglected;
- The last element indicated the probability score of the prediction.

At inference time, only predictions with a probability score greater than a given threshold were selected, and their characteristics were derived from the other four parameters produced by the neural network for each prediction.

### 2.3. Dataset Generation and Training Procedure

A great quantity of data is required to train neural network models, and this is especially true for deep neural networks. The neural network proposed in this paper required a TDR signal such as those described in Section 2.1 as input data and a list of the impedance discontinuity points in the cable as target data. A TL simulator [20] was adopted to produce an adequate number of samples, each of them accompanied by a label containing information about the faults in the cable and the cable length.

#### 2.3.1. Simulation Procedure

**Figure 3:**
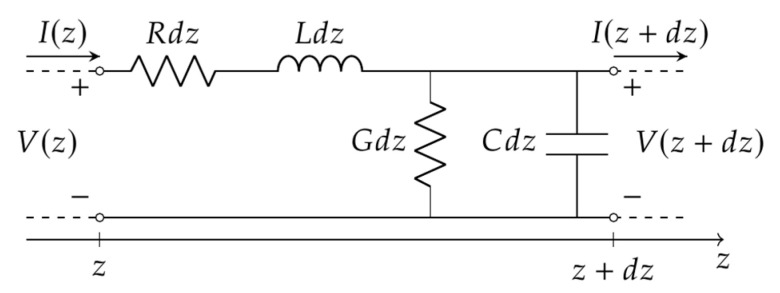
Elementary cell of a transmission line.

Using classical microwave theory, the RG58-CU cable was modeled similarly to a transmission line composed of elementary cells such as that depicted in Figure 3.

**Figure 4:**
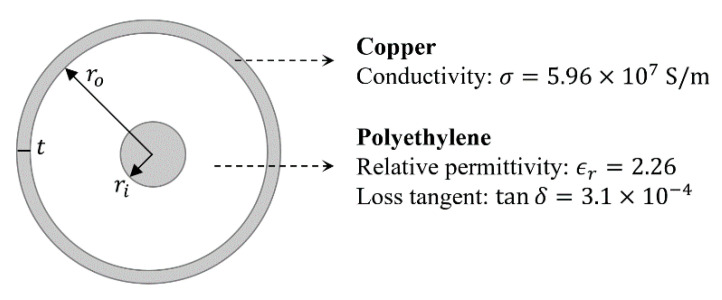
Section of the RG58-CU cable.

The theoretical formulation of the primary parameters of the transmission line was computed considering the dielectric dispersion and the skin effect. The resulting primary parameters were

(2) $$R\left(\omega \right)=\frac{1}{\sigma_{c}\pi r_{i}^{2}}+\frac{1}{\sigma_{c}\pi \left[{\left(r_{o}+t\right)}^{2}-r_{o}^{2}\right]}+\frac{1}{2\pi }\left(\frac{1}{r_{i}}+\frac{1}{r_{o}}\right)\sqrt{\frac{\omega \mu_{0}}{2\sigma_{c}}}$$

(3) $$L\left(\omega \right)=\frac{\mu_{0}}{2\pi }\ln\frac{r_{o}}{r_{i}}+\frac{1}{2\pi \omega }\left(\frac{1}{r_{i}}+\frac{1}{r_{o}}\right)\sqrt{\frac{\omega \mu_{0}}{2\sigma_{c}}}$$

(4) $$G\left(\omega \right)=\omega \tan\delta \frac{2\pi \epsilon_{0}\epsilon_{r}}{\ln(r_{o}/r_{i})}$$

(5) $$C=\frac{2\pi \epsilon_{0}\epsilon_{r}}{\ln(r_{o}/r_{i})}$$

where ω was the angular frequency, $\mu_{0}$ was the vacuum permeability, and $\epsilon_{0}$ was the vacuum permittivity. The other symbols in the equations were the geometrical and electrical parameters of the coaxial cable defined in the scheme of Figure 4 as follows:

- $r_{i}$—Inner conductor ray;
- $r_{o}$—Outer conductor ray;
- t—Outer conductor thickness;
- $\sigma_{c}$—Copper conductivity;
- $\epsilon_{r}$—Insulator relative permittivity;
- tanδ—Insulator loss tangent.

**Figure 5:**
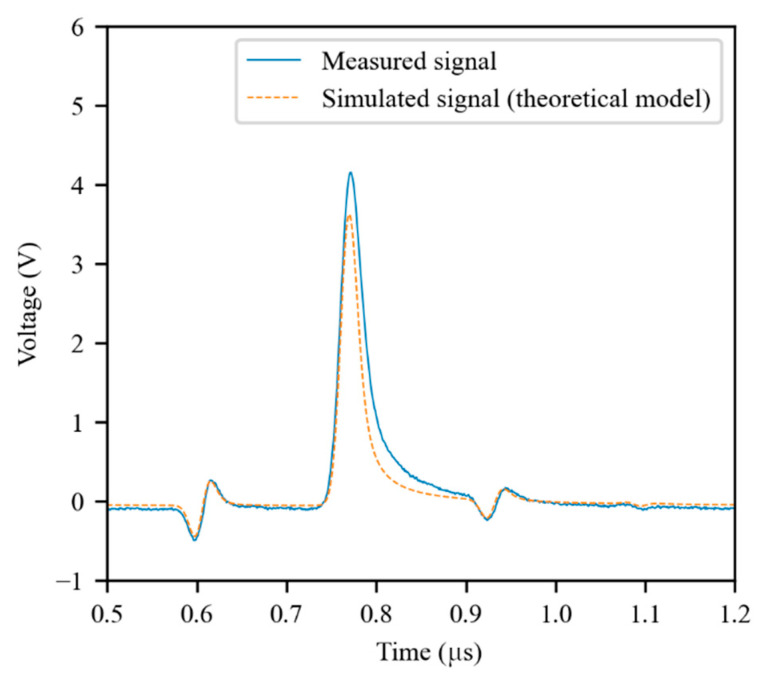
An experimental TDR signal compared with a simulated signal obtained using the theoretical model of the coaxial cable. The cable was 66 m long, with a parallel capacitive fault of 47 pF at 50 m. The first reflected signal is due to the fault, while the second is due to the cable’s open termination.

The effectiveness of the TL model in representing the coaxial cable was evaluated by comparing simulated TDR signals with experimental signals measured in the same configurations. As can be seen in Figure 5, the theoretical model was not able to accurately represent the cable. In particular, the differences in the shape of the reflected signals indicate poor modeling of the frequency-domain behavior of the coaxial cable.

#### 2.3.2. Calibration of the RG58-CU Cable Parameters

A calibration procedure was developed to extract a more accurate model of the primary parameters of the RG58-CU cable. From well-known theory, the propagation function of electromagnetic waves in transmission lines depends on the primary parameters according to the equation

(6) $$\gamma \left(\omega \right)=\sqrt{\left(R+j\omega L\right)\left(G+j\omega C\right)}=\alpha \left(\omega \right)+j\beta \left(\omega \right)$$

As demonstrated in [21], using the SFWR technique, a measure of α(ω) and β(ω) can be obtained for any value of ω. In particular, they can be measured in a range of frequencies of interest and then used to derive an estimate of the primary parameters of the TL in those frequencies.

From (6), the following equation was derived:

(7) $$\gamma^{2}=\alpha^{2}-\beta^{2}+2j\alpha \beta =RG-\omega^{2}LC+j\omega \left(RC+LG\right)$$

Starting from Equation (7), only one pair between R, L and G,C can be estimated using a linear regression technique, e.g., the ordinary least squares (OLS) method. Since the main contribution to the frequency-domain behavior of the TL was derived from the primary parameters R and L, it was decided to estimate them, maintaining the theoretical formulation for G and C instead. The frequency-domain model of R and L was derived from Equations (2) and (3) respectively.

(8) $$R\left(\omega \right)=R_{0}+R_{1}\sqrt{\omega }$$

(9) $$L\left(\omega \right)=L_{0}+L_{1}\frac{1}{\sqrt{\omega }}$$

where $R_{0},\;R_{1},\;L_{0},\;L_{1}$ were the parameters to be estimated with the fitting procedure. Hence, using Equation (7), the following OLS problem was defined:

(10) $$\left[\begin{array}{cccc} G\left(\omega_{1}\right) & G\left(\omega_{1}\right)\sqrt{\omega_{1}} & -\omega_{1}^{2}C\left(\omega_{1}\right) & -\omega_{1}^{2}C\left(\omega_{1}\right)\frac{1}{\sqrt{\omega_{1}}}\;\\ \cdots & \cdots & \cdots & \cdots \\ G\left(\omega_{N}\right) & G\left(\omega_{N}\right)\sqrt{\omega_{N}} & -\omega_{N}^{2}C\left(\omega_{N}\right) & -\omega_{N}^{2}C\left(\omega_{N}\right)\frac{1}{\sqrt{\omega_{N}}}\;\\ \omega_{1}C\left(\omega_{1}\right) & \omega_{1}C\left(\omega_{1}\right)\sqrt{\omega_{1}} & \omega_{1}G\left(\omega_{1}\right) & \omega_{1}G\left(\omega_{1}\right)\frac{1}{\sqrt{\omega_{1}}}\;\\ \cdots & \cdots & \cdots & \cdots \\ \omega_{N}C\left(\omega_{N}\right) & \omega_{N}C\left(\omega_{N}\right)\sqrt{\omega_{N}} & \omega_{N}G\left(\omega_{N}\right) & \omega_{N}G\left(\omega_{N}\right)\frac{1}{\sqrt{\omega_{N}}} \end{array}\right]\cdot [\begin{array}{c} R_{0}\\ R_{1}\\ L_{0}\\ L_{1} \end{array}]=[\begin{array}{c} \alpha^{2}(\omega_{1})-\beta^{2}(\omega_{1})\\ \cdots \\ \alpha^{2}(\omega_{N})-\beta^{2}(\omega_{N})\\ 2\alpha (\omega_{1})\beta (\omega_{1})\\ \cdots \\ 2\alpha (\omega_{N})\beta (\omega_{N}) \end{array}]$$

where $\omega_{i},\;i=1,\ldots ,N$ were the angular frequencies of interest, $G\left(\omega_{i}\right)$ and $C\left(\omega_{i}\right)$ were computed with Equations (4) and (5) respectively, and $\alpha \left(\omega_{i}\right)$ and $\beta \left(\omega_{i}\right)$ were the real and imaginary part of the propagation function of the transmission line measured using the SFWR technique. The N=80 frequencies were linearly spaced in the interval 1–80 MHz, in order to span the entire bandwidth of the stimulus signal. The parameters estimated through the OLS problem are reported in Table 1.

**Figure 6:**
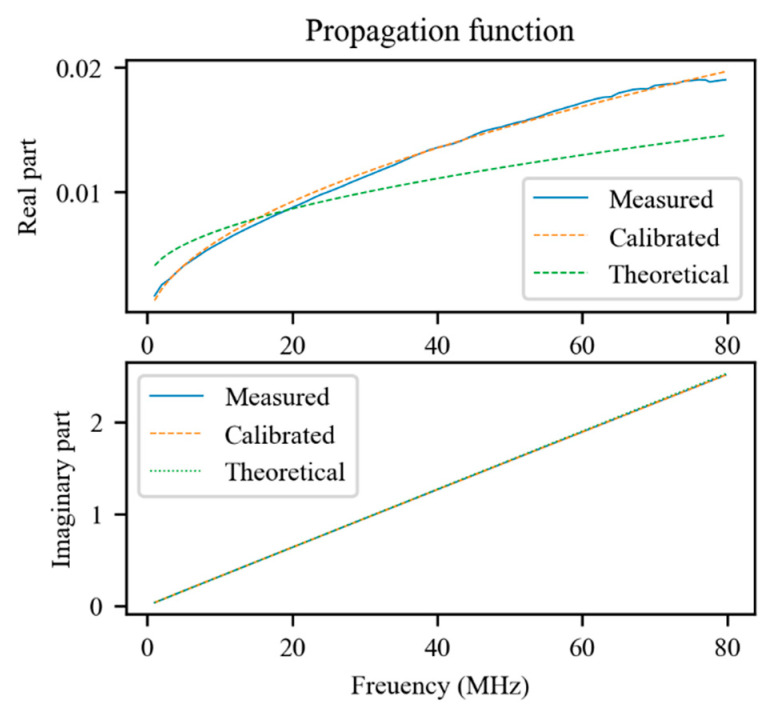
Comparison between the measured propagation function of the transmission line, the theoretical one, and that resulting from the calibration process.

As can be seen in Figure 6, the real part of the propagation function is approximated much better by the model that includes the calibrated primary parameters.

**Figure 7:**
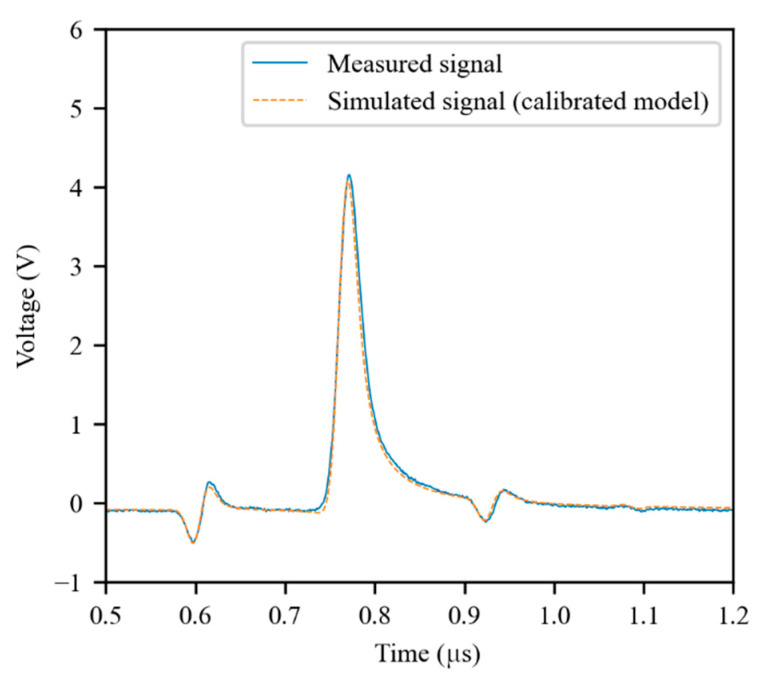
Simulation obtained using the calibrated model for the same configuration of Figure 5.

As expected, the simulator produces much more accurate results when the calibrated primary parameters are used. As an example, the same configuration of Figure 5 was simulated using the calibrated parameters. The result of the simulation is presented in Figure 7. As can be seen, there is a much better fitting between the measured signal and the simulated one. The global root-mean-squared error (RMSE) for all the experiments described in Section 3.2 was 0.098 V using the theoretical model, while it was 0.044 V using the calibrated model. Therefore, the RMSE was more than halved owing to the calibration process.

#### 2.3.3. Dataset Generation

The dataset for training the neural network was therefore generated using the calibrated RG58-CU cable model. Simulations were carried out using the setup described in Section 2, considering the constraints reported in Table 2 to randomly generate the circuital configuration of each sample.

A total of $10^{6}$ samples were generated. After simulations, the TDR signals were processed to remove the transmitted pulse, which did not provide any information given that the signals were time aligned, and to add Gaussian noise. Labels for all the samples were defined in compliance with the format of the neural network’s output. The range of lengths that contained discontinuity points (faults and line terminations) was divided into S=32 cells, and the positions of the discontinuity points $z_{i}$ were normalized relative to the range of lengths of the corresponding cells, obtaining values $0\leq z_{i}^{\prime }<1$. For each sample, an array p of length S was defined whose elements were 1 if the corresponding cell contained a discontinuity point and 0 otherwise. The class of each discontinuity point (fault or line termination) was specified through a S×2 matrix that had elements $c_{m,n}=1$ for m corresponding to cells containing a discontinuity point and n corresponding to the class of the discontinuity point (1 for faults, 2 for line termination) and 0 otherwise. Finally, the capacities associated with the faults, $C_{i}$, were normalized relative to the range reported in Table 2, computing the values $C_{i}^{\prime }$.

#### 2.3.4. Training of the Neural Network

The CNN described in Section 2.2 was implemented in Python, using the TensorFlow library. In total, 80% of the samples of the dataset were used for neural network training, while the remaining 20% were used for validation. In the training procedure, the following multi-objective loss function was minimized:

(11) $$L=\alpha_{1}\frac{1}{N_{f}+1}\;\overset{S}{\underset{n=1}{\sum }}p_{n}{\left({\hat{z}}_{n}^{'}-z_{n}^{'}\right)}^{2}+\frac{1}{2(N_{f}+1)}\;\overset{2}{\underset{m=1}{\sum }}\overset{S}{\underset{n=1}{\sum }}p_{n}{\left({\hat{c}}_{m,n}-c_{m,n}\right)}^{2}+\alpha_{2}\frac{1}{N_{f}}\overset{S}{\underset{n=1}{\sum }}p_{n}c_{1,n}{\left({\hat{C}}_{n}^{'}\;-C_{n}^{'}\right)}^{2}\;+\frac{1}{N_{f}+1}\overset{S}{\underset{n=1}{\sum }}p_{n}{\left({\hat{p}}_{n}-p_{n}\right)}^{2}+\frac{1}{S-(N_{f}+1)}\overset{S}{\underset{n=1}{\sum }}(1-p_{n}){\left({\hat{p}}_{n}-p_{n}\right)}^{2}\;$$

where the estimates produced by the neural network were marked with a hat. The parameters $\alpha_{1}=50$ and $\alpha_{2}=100$ were introduced as a result of a tuning procedure that involved the maximization of the performances of the neural network on the validation dataset.

The neural network model was trained for 300 epochs using the Adam optimizer [22]. The learning rate was varied according to exponential decay, updating its value at the end of each epoch so that it halved every 200 epochs.

The weights of the neural network model after the training procedure, as well as the training and validation datasets of simulated signals and the test dataset of experimental signals presented in Section 3.2, are available in [23].

## 3. Results and Discussion

The performances of the CNN were assessed considering both simulated and experimental signals. The threshold for the probability score of predictions was set to half the range (0.5). A discontinuity point prediction was considered correct if a discontinuity point of the same class was present in the label at distance less than the length of a cell. A reflectometry signal was therefore considered correctly analyzed if all the faults were detected without any false positive.

### 3.1. Performance Assessment on the Validation Dataset

The performance of the neural network was first assessed using a validation dataset that was composed of $2\times 10^{5}$ samples. The outputs produced by the neural network were compared with the corresponding labels. Overall, 99.87% of simulated cables were correctly assessed, meaning that all the faults were correctly identified, and there were no false positives. Estimation errors for the correctly detected discontinuity points were computed and are summarized in Table 3. The RMSE in the estimation of discontinuity points (both faults and line terminations) was below 10 cm, leading to a mean absolute percentage error (MAPE) lesser than 0.1%. The MAPE in the capacity estimation was below 2% instead.

### 3.2. Performance Assessment on the Test Dataset (Experimental Data)

The same kind of assessment was performed considering experimental data. In this case, 100% of the cables were correctly characterized. Estimation results for experiments on cables with one, two, three, and four simulated capacitive faults are reported, respectively, in Table 4, Table 5, Table 6 and Table 7.

The results of the statistical analysis of the estimation errors obtained for all the experiments are reported in Table 8. The errors obtained for experiments are slightly greater than those obtained for the validation simulated samples; however, good accuracy was obtained in both position and capacity estimation.

## 4. Conclusions

In this study, a method to detect and characterize impedance discontinuity points in cables was presented. The method, based on the use of TDR and CNNs, was characterized for the case of capacitive faults in coaxial cables, but it can be used in any situation where localized impedance discontinuity points are present. In fact, only retraining of the neural network with an appropriate dataset of labeled reflectometry signals would be required for the neural network to work in a different condition. Typical applications for the proposed method are the localization and characterization of multiple discontinuity points in a distributed sensing element, e.g., faults in cables, leaks in pipelines, damages in concrete structures. The metrological characterization of the method presented in this paper, revealed by analyzing the estimation errors for both simulated and actual experimental signals, proves that it can localize the discontinuity points with great accuracy.

## Figures and Tables

**Table 1:** Estimates of the parameters of R(ω) and L(ω) models.

$R_{0}\;\left(\Omega \cdot m^{-1}\right)$	$R_{1}\;\left(\Omega \cdot m^{-1}\cdot Hz^{-1/2}\right)$	$L_{0}\;\left(H\cdot m^{-1}\right)$	$L_{1}\;\left(H\cdot m^{-1}\cdot Hz^{1/2}\right)$
0.022	$6.7\times 10^{-5}$	$2.3\times 10^{-7}$	$7.5\times 10^{-5}$

**Table 2:** Parameters of the simulated transmission lines.

	Number of Faults $N_{F}$	Distance between Discontinuity Points (m)	Total Length of the Cable (m)	Capacity of the Faults (pF)
**Min**	0	10	10	50
**Max**	4	-	200	500

**Table 3:** Estimation errors obtained for the validation dataset.

Cable Length Error		Fault Position Error		Fault Capacity Error	
RMSE (m)	MAPE	RMSE (m)	MAPE	RMSE (pF)	MAPE
0.070	0.059%	0.066	0.091%	11	1.2%

**Table 4:** Estimation results for real cables with one capacitive fault.

Position of the Fault (m)		Capacity of the Fault (pF)		Length of the Cable (m)	
Nominal	Estimated	Nominal	Estimated	Nominal	Estimated
50	49.94	107	112	65	65.00
50	49.92	152	158	65	65.01
50	49.97	217	205	65	64.92
50	49.92	309	310	65	64.95
50	49.96	404	434	65	65.01
50	49.99	450	450	65	64.96

**Table 5:** Estimation results for real cables with two capacitive faults.

Position of Fault 1 (m)		Capacity of Fault 1 (pF)		Position of Fault 2 (m)		Capacity of Fault 2 (pF)		Length of the Cable (m)	
Nominal	Estimated	Nominal	Estimated	Nominal	Estimated	Nominal	Estimated	Nominal	Estimated
15	15.04	107	112	65	65.10	152	156	81	81.05
15	15.04	107	113	65	65.21	217	206	81	80.97
15	15.05	107	112	65	65.18	450	451	81	80.80
15	15.13	217	206	65	65.03	309	309	81	80.83
15	15.12	217	206	65	65.02	404	433	81	80.84
15	15.14	450	459	65	65.14	404	430	81	80.69

**Table 6:** Estimation results for real cables with three capacitive faults.

	**Experiment 1**		**Experiment 2**	
	**Nominal**	**Estimated**	**Nominal**	**Estimated**
**Length of the Cable (m)**	131	130.99	131	130.93
**Position of Fault 1 (m)**	50	49.90	50	49.93
**Position of Fault 2 (m)**	65	65.06	65	64.99
**Position of Fault 3 (m)**	115	115.09	115	114.92
**Capacity of Fault 1 (pF)**	107	115	217	214
**Capacity of Fault 2 (pF)**	217	210	450	454
**Capacity of Fault 3 (pF)**	404	431	404	418

**Table 7:** Estimation results for real cables with four capacitive faults.

	Experiment 1		Experiment 2	
	Nominal	Estimated	Nominal	Estimated
**Length of the Cable (m)**	143	142.96	143	142.96
**Position of Fault 1 (m)**	50	49.87	50	49.88
**Position of Fault 2 (m)**	65	65.12	65	65.03
**Position of Fault 3 (m)**	115	114.93	115	114.95
**Position of Fault 4 (m)**	131	131.40	131	131.32
**Capacity of Fault 1 (pF)**	107	117	107	115
**Capacity of Fault 2 (pF)**	217	214	152	165
**Capacity of Fault 3 (pF)**	404	436	309	323
**Capacity of Fault 4 (pF)**	450	441	450	439

**Table 8:** Estimation errors obtained for experimental signals.

Cable Length Error		Fault Position Error		Fault Capacity Error	
RMSE (m)	MAPE	RMSE (m)	MAPE	RMSE (pF)	MAPE
0.12	0.10%	0.13	0.22%	14	4.3%

## Data Availability

The data presented in this study are openly available in Zenodo at doi:10.5281/Zenodo.5720438, reference number [23].
